# The Diagnosis of Typhoid Fever

**Published:** 1907-09-21

**Authors:** 


### THE DIAGNOSIS OF TYPHOID FEVER.

PROFESSOR CHANTEMESSE, the discoverer of a typhoid serum, has communicated to the French Academy of Medicine a means of diagnosing typhoid fever somewhat similar to that recently adopted by Dr. von Pirket, of Vienna, and Dr. Wolff-Eisner for the detection of tuberculosis. In the latter case a drop of tuberculin introduced into the eye of a person affected with tuberculosis produces a reaction which is not observable in healthy subjects, and Professor Chantemesse directed his experiments with the view of discovering whether the poison of typhoid fever similarly employed would indicate as clearly and certainly the presence of typhoid fever. The toxin is prepared in the form of a powder, one-fiftieth of a milligramme of which is dissolved in a drop of water, and placed under the eyelid, when a clear proof of the presence or absence of typhoid fever is given without inconvenience or danger. Neither the temperature nor the general condition of the body is affected by this test. When typhoid fever is present the eye becomes bright red, and a flow of tears is produced for several days, while, if it is not present, these symptoms are far less marked and disappear in the course of a few hours. Professor Chantemesse declares as the result of many experiments that this method is absolutely without danger, while the certainty of the diagnosis will put an end to the difficulty experienced so frequently of deciding whether a patient is suffering from typhoid fever or another disease presenting many of the same symptoms.

